# Experiences of the parents caring for their children during a tuberculosis outbreak in high school: a qualitative study

**DOI:** 10.1186/1471-2458-14-132

**Published:** 2014-02-07

**Authors:** Shaoru Zhang, Wei Ruan, Yingqun Li, Xiangni Wang, Xing Wang

**Affiliations:** 1 Department of Nursing, Xian Jiaotong University, 76 Yanta West Road, Postbox 38, Xian City, Shaanxi Province 710061, China

**Keywords:** Tuberculosis, High school students, Parents, Care burden, Qualitative research

## Abstract

**Background:**

Tuberculosis (TB) remains a serious epidemic in China. In the past five years, the number of TB infections in high school students is rising and thus high school students are becoming a high risk group of TB. Parents of children with TB have to endure high psychological pressures from the disease itself, children’s education, employment and life. The purpose of this study was to investigate the psychological pressure of parents with high school students suffering from TB.

**Methods:**

A total of 22 parents who have been taking care of their children suffering from TB were interviewed and a framework approach was used to analyze the interviews.

**Results:**

In our study, 21/22 parents had low levels of understanding about TB; 22/22 were under psychological stress; and 20/22 stated that their daily life was impacted on TB.

**Conclusions:**

Parents need to be given appropriate knowledge on TB and psychological counseling. Authorities should not only implement the therapeutic measures, but also focus on solving the psychological problems of patients and their families when a similar outbreak occurs.

## Background

Tuberculosis (TB) is a chronic infectious disease that seriously affects human health and daily life worldwide [1]. China is one of the 22 countries with high occurrence of TB, and has the world’s second highest number of TB cases, accounting for about 12% of total cases throughout the world [2]. Students have become a high risk group of TB [3]. In 2010, the national network direct reporting system reported that there were 48,961 students suffering from TB, which accounted for 4.94% of the total TB cases. In many Provinces of China, the number of students with TB ranked second just behind farmers and herdsmen. TB that occurred in students with the age of 15–24 accounted for 83.4% of the total students with TB [4]. Clusters of cases or outbreaks of TB have occurred nationally in high schools [5-7], which not only affects the normal teaching order, but also brings a lot of trouble to the families. Parents of students with TB have to endure pressures from the disease itself, children’s education, employment and life and their own psychological issues. However, many studies only focus on on the psychological problems and intervention approaches of students with TB [8,9]. There are no reports on the experience of parents caring for their children with TB.

In March 2013, two cases of active TB students were reported in a high school located in the northern part of Shaanxi Province. The cases came to the close attention of the health department. The tuberculosis Prevention Institute of Shaanxi Province promptly organized an emergency response team to conduct the TB screening for all the students in three local high schools and a total of 22 cases of active tuberculosis among students were found. These 22 students were immediately isolated to the local hospital for chemotherapy. Under the guidance of the ERT, the logistics departments in the three schools disinfected the dormitories, classrooms and dining rooms. In this study, we interviewed parents of students with TB and expected to identify the difficulties that were faced during the caring process of their children. As a communication bridge between local Center for Disease Control (CDC) and parents, researchers in this study provided emotional and information support through the in-depth interviews.

## Methods

### Design

All the data in this study was qualitative and collected by in-depth interview. In March 2013, two cases of active TB students were successively identified in a high school in the county of northern Shaanxi Province. TB researchers worked with the Tuberculosis Prevention Institute of Shaanxi Province to conduct a comprehensive screening for TB, and provided health education for high school students. Meanwhile, practical problems were discovered and solved through individual in-depth interviews.

### Interviewers

There were two interviewers. One is a professor of the Xian Jiaotong University School of Medicine. The professor has participated in the qualitative research conferences organized by Australian experts and had extensive qualitative research experience. In addition, this professor also had extensive interviewing experience with acquired immune deficiency syndrome (AIDS), tuberculosis, school medical stuff and the staff of TB preventive institute. The second professor was from Tuberculosis Hospital of Shanxi Province. The professor had been engaged in the diagnosis and treatment of TB for a long time and had a wealth of experience in clinical practice. In this study, the second professor is responsible for TB screening, diagnosis and interviewing work. Two medical graduate students who received trainings on data collections for qualitative researches were responsible for recording the interviews.

### Participants

With the assistance of local CDC, we performed in-depth interviews with the parents of 22 students with TB in the high school of Shanxi Province. The demographic characteristics of the parents are shown in Table 1.

**Table 1:** Demographic characteristic of the participants

	**Gender**		**Educational level**			**Native place**		**Student (years in the high school)**		
	**Male**	**Female**	**MS**	**HS**	**C**	**Countryside**	**Urban**	**1**^**st **^**year**	**2**^**nd **^**year**	**3**^**rd **^**year**
Number	14	8	14	4	4	13	9	6	7	9
(%)	63.6	36.4	63.6	18.2	18.2	59.1	40.9	27.3	31.8	40.9

MS: High school; HS: High school, C: College.

### Data collection

In March 2013, we performed Zarit Burden Interview (ZBI) as described previously [10] to measure the parents’ (caregivers) personal strain, role strain and total ZBI. In the 1980s, American scholar Zarit gave the definition to care burden, and designed the ZBI scale. As an effective tool, the ZBI was widely used for measuring the burden of caregivers by domestic and overseas researchers. The scale is composed of 22 items, including personal strain and role strain. The ZBI asks caregivers questions about physical, psychological, economic, and communication problems that cause stress and strain for caregivers. The items are answered on a five-point scale ranging from 0 (never) to 4 (always). Scores were calculated by summing up the total chosen statement which ranges from 0 to 88, that higher scores implying greater perceived caregiver burden. If the final score is less than 39 points, it means the caregiver bears the low level of the burden; If the score is between 40 to 59 points, it means the caregiver bears the burden of medium level; If the score is greater than 60 points, it means the caregiver bears the heavy burden level. The Chinese scholars Wang Lie translated the ZBI into Chinese in 2006, and it has good validity according to the research (Cronbach’ α = 0. 91, ICC = 0.71, r = 0.41-0. 71). The Chinese version ZBI is widely used in China.

After the parents completed the scale, the researchers performed personal in-depth semi-structured interviews to collect data based on the voluntary, confidential, and convenient principle. The interviewer first introduced the purpose, method, the confidential principle and the necessity of recording of the interview. The interview started after obtaining the informed consent from the interviewees and their parents. Parents of 16 students took part in the interviews in a single room of the infectious department at a local county hospital and parents of the other six students received interview in an independent office of the TB preventive institute. The interviewing place was quiet and private. Interviews included the experiences of TB treatment, psychological and life impact of TB and concerns on the students’ studies. Each interview usually lasted for 35–50 minutes. The interview outline is shown in Table 2.

**Table 2:** Interview outline

1	How was the disease discovered and what are the possible causes of the disease
2	What are the biggest problems for the students with TB (time, energy or pressure)?
3	What is the biggest concern?
4	How do you look at the arrangement of the treatment and deferment? If it is not reasonable, what is your thought on the arrangement?
5	What is the impact of the children’s health insurance on the family’s expenses?
6	What do you know about TB? (including knowledge on 7 core information)

### Ethical considerations

This study was approved by Ethics Committee of Xian Jiaotong University, School of Medicine. TB is a contagious disease. Patients and their families had varying degrees of stigma. Thus, in view of ethical considerations, all participants were assured that their identities would be kept confidential.

### Data analysis

Analysis of the data followed the “framework” approach. The first step of the analysis was to transcribe the audio-record, word for word. The researcher listened repeatedly to the audio-record and read the noted observations to correct the transcripts. After repeatedly reading the transcripts, a code frame was progressively established based on recurring viewpoints emerging from the data and the interview guideline. Every transcript was then coded systematically against the code frame. Codes were merged into categories and then these categories were organized into themes. Disagreements were discussed among the research team to reach a final consensus. The principal researcher revisited the main points of the findings with the participants and asked whether they were consistent with their experiences. The process of analysis was conducted in Chinese and the results were translated to English.

We used SPSS19.0 statistical software package to analyze the data from the ZBI questionnaire. We used descriptive statistics, independent sample t-test, analysis of variance of statistical method to analyze their burden level, with P < 0.05 for the statistically significant difference.

## Results

The ZBI scale assessment showed that personal burden and roles of the score was (30.32 ± 6.92) and (11.23 ± 5.19), respectively. The total burden score was (51.50 ± 12.73). The personal burden score was significantly higher than the role of score (p < 0.01), indicating that the parents (caregivers) had significant anxiety, stress and other psychological feelings. There were 18 parents (82%) under the moderate or severe burden of care and a few parents had minor burden of care (Table 3). The results also showed that the burden of parents had no differences in gender (t = 0.44, p > 0.44), degree of culture (F = 0.7, p > 0.7) and the Native place (P > 0.24), which may be due to the small sample size included in this study (Table 4).

**Table 3:** Zarit burden interview

	**Severe**	**Moderate**	**Mild**
	**(ZBI score: 60~)**	**(ZBI score: 40~59)**	**(ZBI score: ~39)**
Number of parents	5	13	4
(%)	22.7	59.1	18.2

**Table 4:** Statistical description about burden and general information

	**Gender**		**Educational level**			**Native place**	
	**Male**	**Female**	**MS**	**HS**	**C**	**Countryside**	**Urban**
Number	14	8	14	4	4	13	9
(%)	63.6	36.4	63.6	18.2	18.2	59.1	40.9
Score of burden	50.57 ± 11.75	53.13 ± 15.00	53.43 ± 14.42	44.75 ± 6.95	51.50 ± 10.38	50.92 ± 12.30	52.33 ± 14.05
t/F	-0.44		0.70			-0.24	
P	>0.05		>0.05			>0.05	

MS: High school; HS: High school, C: College.

After the interview data were analyzed, three main findings were obtained: (1) the parents lack appropriate knowledge on TB; (2) the parents had high psychological pressure; and (3) the daily life of the parents was impacted (Table 5).

**Table 5:** Categories and subcategories the data derived from interview

**Categories**	**Subcategories**	**Number of patients (%)**
High psychological pressure	Fear of disease treatment	22 (100)
	Worried about their education	
	Worried about prognosis	
	Negative emotions	
Impacted daily life	Interruption of the normal work	20 (90.1)
	Increased economic burden	
	Lack of energy	
Lack of knowledge	Weak awareness of TB	21 (95.5)
	Lack of knowledge of disease	
	Lack of policy information	

### High psychological pressure

The parents were mostly concerned about the treatment, prognosis and the impact on the students’ education. Thus, they had a high psychological pressure with a negative emotion.

#### Worries about the disease treatment

Most of the parents were concerned about the treatment of their children. They actively cooperate with doctors to provide nutritional support and expect a speedy recovery. The following two examples were parents’ response during the interview.

“I heard that this is a serious disease and cannot be easily cured. My concern is if this disease can be completely cured” (Mrs. S, Age: 44).

“What I am worried is the child’s disease situation. I want to provide extra nutrition support to my child because health is the most important” (Mrs. L, Age: 40).

#### Worries about the children’s education

Most of the students are facing national entrance exams to college. Infection with TB at this time will definitely affect their performance in the exams. The parents worried about the interruptions of their children’s studies. The following three examples were parents’ response during the interview.

“This is a big pressure for us. The child is now in the hospital and cannot keep up with his high school studies” (Mr. W, Age. 48).

“The pressure for us is that my child cannot go to school now. There will be the national entrance exam to college very soon, so we are very anxious” (Mr. C, Age: 44)

“My son is now in the third year of high school, and has a very good performance in his studies before the disease. TB will definitely affect his performance in the exams” (Mr. W, Age: 44).

#### Worries about the prognosis

Parents were anxious that their children will have tuberculosis sequelae, and worried that the disease will create an obstacle for future employment. The following two examples were parents’ response during the interview.

“What I am most worried about is the sequelae of TB. I am afraid that my child will not pass the medical exam to go to the college” (Mr. Z, Age: 43)

“My daughter had not been in a good health condition. Now she had TB, I am worried it would be difficult for her to find a job in the future” (Mr. W, Age: 48).

#### Negative emotions

Because the parents are faced with many difficulties, they described their worries and anxieties. Some parents even do not know how to deal with this situation and have silent tears. The following two examples were parents’ response during the interview.

“I worried about this every day” (Mr. W, Age: 49).

“I do not know what to do. The child is not feeling well and I am very worried about him, but cannot say too much” (Mr. W, Age: 48).

### The daily life is affected

The parents’ daily life was disrupted. Caring of their children becomes the main daily task.

#### Interruption of daily work

The parents who need to go to work every day have to call personal leave to take care of their children in the hospital, which severely affects their normal life. The following two examples were parents’ response during the interview.

“I work in the field. My child is sick and I have to stop my job to take care of my child” (Mr. W, Age: 44).

“I am a driver. My child is hospitalized and I have to call personal leave” (Mr. Z, Age: 48).

#### Increased economic burden

Cost for the disease treatment and reduced income due to work interruptions increases the family’s economic burden. Some families have to borrow and loan money to maintain the treatment for their children. The following two examples were parents’ response during the interview.

“Economic issue is the most difficult issue for us” (Mr. W, Age: 44).

“My child is sick and we have to borrow money to treat the disease. When the disease is cured, our economic burden will be gone” (Mrs. L, Age: 39).

#### Lack of energy

Because of the caring for the hospitalized children, many parents have no time to take care of the elderly and other children. A lack of time and energy was reported by the parents. The following two examples were parents’ response during the interview.

“I have another 5-year old kid at home and I do not have time to take care of him because of the hospitalized child” (Mrs. L, Age: 45).

“I have a 80-year old elderly person to take care of and it is difficult to care of both hospitalized child and the elderly person. I feel short of time and energy” (Mr. C, Age: 42).

### Lack of knowledge

After the TB cases were discovered in the students, parents still did not fully understand the disease. There was a lack of knowledge about TB among the parents. This lack of knowledge resulted in the parents displaying more passive behavior in relation to the treatment and prevention of TB.

#### Lack of awareness of TB

Parents did not pay attention to the occurrence of TB cases on campus. Parents also did not urge students to have a medical examination. Parents of six cases (27%) who had symptoms of coughing and fever were still unaware of timely treatment. A total of 18 cases (81%) were identified by the TB screening, which was conducted by schools in response to the situation, and only four cases (18%) visited hospital as a result of symptoms. Therefore, early control of the epidemic was delayed. The following two examples were parents’ response during the interview.

“My daughter had cough and lost 12 pounds in a week. I was informed that she had TB after school medical examination” (Mrs. G, Age: 41).

“My son began with a cold and runny nose. X-ray examination by the school found that he had TB” (Mrs. L, Age: 40).

#### Lack of knowledge on TB

After diagnosis, parents of 13 cases (59%) still did not understand the main route of TB transmission and basic preventive measures. Parents of 15 cases (68%) lack the knowledge of the typical symptoms of TB. Parents of 19 cases (86%) did not have a clear understanding of the TB treatment. The following two examples were parents’ response during the interview.

“No one at home has ever had TB, and we do not understand this disease” (Mr. Z, Age: 48)

“I do not receive much education and I do not know how much time is required to cure TB” (Mr. W, Age: 44).

#### Lack of policy information

Parents of only four TB cases (18%) knew the policy of TB territorial management. The TB territorial management requests that the TB patients of students should register in the school district CDC, accept the treatment and management. The parents of half of the TB cases did not understand the free-medical policy, including free examination and free medicine. The following three examples were parents’ response during the interview.

“My daughter had TB and nobody told us where she can receive treatment” (Mrs. L, Age: 40)

“We do not fully understand the medical reimbursement policy of TB treatment and we do not know whom we should ask about these issues” (Mrs. G, Age: 41)

“I heard there are free medical examination items, I was not clear what these items are” (Mrs. L, Age: 45)

## Discussion

The identification of TB cases in the high school resulted in much stress and anxiety for many parents. Based on the interview and the ZBI score, we found that parents had high psychological pressures. Many parents had anxious emotions and choked with sobbing during the interview. The parents were mostly worried about the children’s health and disrupted high school studies. The majority of the parents hoped that the children did not miss the studies during the treatment, which is particularly evident for the parents of more senior students in the high school. According to the TB control and treatment guidelines, the contagiousness of TB disappears after 2–3 weeks of regular treatment, and the patients can participate in social activities. However, in order to prevent the spread of the epidemic and avoid the adverse emotions of other students and their parents, most schools require students to be completely cured before returning to school [11]. Thus, the students face the situations of missing studies in the school. Yet, it is well known that the national college entrance exam is very competitive and is the most important event for the entire family. The most critical studying period for the exam is in the high school [12]. Studies are missed and the students’ performance in the national exam will be impacted due to the TB treatment. Thus, the students with TB may not be accepted to the ideal university. On the other hand, considering the importance of the national exam, the school may allow some non-contagious students to continue their studies in the school. However, studying in the school during the period of oral drug therapy, will lead to insufficient rest. Thus, the parents face a dilemma: having rest at home or continuing to study at school.

In addition, China has a saying of “ten TB cases, nine dead”, which deeply influences people’s thought [13]. People are still in fear of TB. What the parents are mostly concerned is whether or not TB can be cured. Parents are worried that inadequate treatment of the disease affects the medical qualification for the college entrance. They are afraid that the disease history of TB may become an obstacle of their children’s future education and employment [14]. Also, because TB is contagious, people have a prejudice against TB patients [15]. Psychological pressure of the students and the parents also comes from the alienation and even discrimination from their friends [16]. Therefore, they use negative approaches, e.g., self-isolation and concealing of the disease to reduce the adverse effects. However, hiding disease because of stigma may lose the help from their relatives and the community and is not conducive to the prevention and control of TB.

Caring activities also increase the burdens of the parents. After their children get TB, parents have to adjust their working time and reduce their entertaining activities in order to take care of their children in the hospital. Currently, China is in the “family planning policy”, the number of children in a family is smaller. Parents take much care of each child. Meanwhile, the performance in the college entrance exam is the most important criteria to evaluate and select talented persons. The school often neglects the cultivation of students’ comprehensive capability [17]. The high school students generally have poor self-care ability. Therefore, parents have to take care of their children in hospital. On the other hand, under the influence of Confucianism, traditional families follow the intergenerational co-residence structure [18]. The parents of students are under the double burden of taking care of their children and the elderly persons. Therefore, parents often feel everywhere at once and lack of both time and energy.

During the investigation, we found that parents of the students lacked the knowledge on TB. Other Studies have shown that awareness on the prevention of infectious disease is relatively low [19]. When the suspicious symptoms such as cough, hemoptysis appeared, they cannot be aware of TB infection. In recent years, active public education of TB has been enriched and expanded [20]. Due to the lack of interest in the knowledge about TB, TB prevention and control is difficult [21]. Lack of the knowledge on TB symptoms, territorial management and free policies often miss the best timing of treatment, leading to delays in diagnosis of the disease [22]. Most of the interviewees in this study are from rural areas. They have low educational level and weak comprehension ability. Coupled with the occlusion of information channels, parents learn TB mainly from the misconceptions of the family members or relatives [23]. The parents cannot provide guidelines to the children for prevention of TB. Medical screening, diagnosis and treatment are usually a passive process. This study also found that only 5 parents (5/22) thought that the disease brought negative economic issues. After the outbreak of TB, local government has committed to the implementation of cost-free policy for patients. However, some families are still facing temporary economic difficulties due to the medical cost before reimbursement, extra nutrition support and decreased income caused by reduced working time.

Our suggestion has a profound significance for future research. It can be a reference for the government to make the support policy. In the follow-up study, we will be conducting a similar study in the city’s parent group, discussing differences of care experience between urban and rural parents. At the same time, we will focus on the healthy education and family counseling as the main research direction, in order to identify the new mode of response to infectious diseases.

## Conclusions

In this study, we obtained a deeper understanding of the actual situations faced by parents of students with TB through field investigation and personal interview. We also solved many problems immediately for the families. Relevant policies of TB treatment to parents are required, in order to strengthen the confidence of parents to prevent TB. Being as a bridge connecting patients with local CDC, we reflected the family economic difficult problem to the local CDC, prompting the local health department to carry out the policy of free of charge during the treatment in hospital. This largely reduced the economic burden of parents.

Although parents face many difficulties, they can still make self-adjustment and provide the best care for their children. When a similar outbreak occurs in the future, authorities should not only implement the treatment measures, but also focus on solving the psychological problems of patients and their families. The authorities should also provide information resources to prevent the spread of TB.

## Abbreviations

TB: Tuberculosis; CDC: Center for Disease Control; AIDS: Acquired immune deficiency syndrome; ZBI: Zarit Burden Interview.

## Competing interests

The authors declare that they have no competing interests.

## Authors’ contributions

SZ designed the study in collaboration with WR and YL. WR coded the data and all authors contributed in the subsequent analysis. SZ and WR drafted the manuscript. All authors have critically revised the manuscript. All authors read and approved the final manuscript.

## Authors’ information

Shaoru Zhang is a professor in the College of Medicine at the Xian Jiaotong University. Her main research fields are the primary health care, infectious disease and public health.

Wei Ruan is a Master’s student of the Department of Nursing, Xian Jiaotong University. Ruan is specializing in public health nursing and her research topic is tuberculosis prevention among undergraduates.

Yingqun Li, Xiangni Wang and Xing Wang are Master’s student of the Department of Nursing, Xian Jiaotong University. They are specializing in public health nursing.

## Pre-publication history

The pre-publication history for this paper can be accessed here:

http://www.biomedcentral.com/1471-2458/14/132/prepub
